# Low-dose ionizing radiation exposure represses the cell cycle and protein synthesis pathways in *in vitro* human primary keratinocytes and U937 cell lines

**DOI:** 10.1371/journal.pone.0199117

**Published:** 2018-06-18

**Authors:** Kazumasa Sekihara, Kaori Saitoh, Haeun Yang, Haruki Kawashima, Saiko Kazuno, Mika Kikkawa, Hajime Arai, Takashi Miida, Nobuhiro Hayashi, Keisuke Sasai, Yoko Tabe

**Affiliations:** 1 Department of Laboratory Medicine, Juntendo University Graduate School of Medicine, Tokyo, Japan; 2 Leading Center for the Development and Research of Cancer Medicine, Juntendo University Graduate School of Medicine, Tokyo, Japan; 3 Deparmentof Life Science and Technology, Tokyo Institute of Technology Graduate School of Bioscience and Biotechnology, Tokyo, Japan; 4 Laboratory of Proteomics and Biomolecular Science, Research Support Center, Juntendo University Graduate School of Medicine, Tokyo, Japan; 5 Department of Radiation Oncology, Juntendo University Graduate School of Medicine, Tokyo, Japan; 6 Department of Next Generation Hematology Laboratory Medicine, Juntendo University Graduate School of Medicine, Tokyo, Japan; Northwestern University Feinberg School of Medicine, UNITED STATES

## Abstract

The effects of the high-dose ionizing radiation used in radiotherapy have been thoroughly demonstrated *in vitro* and *in vivo*. However, the effects of low-dose ionizing radiation (LDIR) such as computed tomography-guided biopsies and X-ray fluoroscopy on skin cells remain controversial. This study investigated the molecular effects of LDIR on the human primary keratinocytes (HPKs) and U937 cells, monocytes-like cell lines. These cells were exposed to 0.1 Gray (Gy) X-ray as LDIR. The modulation of transcription was assessed using a cDNA array, and the protein expression after LDIR exposure was investigated using isobaric tags for relative and absolute quantification (iTRAQ) proteomic analysis at 24 hours. These effects were confirmed by immunoblotting analysis. The direct effects of LDIR on the U937 cells and HPKs and the bystander effects of irradiated HPKs on U937 cells were also investigated. LDIR downregulated c-Myc in both U937 cells and HPKs, and upregulated the p21^WAF1/CIP1^ protein expression in U937 cells along with the activation of TGFβ and protein phosphatase 2A (PP2A). In HPKs, LDIR downregulated the mTOR signaling with repression of S6 and 4EBP1 activation. Similar changes were observed as bystander effects of LDIR. Our findings suggest that LDIR inhibits protein synthesis and induces the cytokines activation associated with inflammation via direct and bystander effects, which might recapitulate the effects of LDIR in inflammated skin structures.

## Introduction

Epidemiological and toxicological studies have examined the deleterious effects of radiation exposure in human health. High-dose ionizing radiation clearly causes harmful consequences for humans. By contrast, the risk of low-dose ionizing radiation (LDIR) is still unclear. The stochastic risk of LDIR (*i*.*e*., <0.1 Sv) is derived by extrapolation from data obtained for high-dose radiation exposure using a linear-no-threshold (LNT) model [1]. A recent epidemiological study demonstrated that the data from atomic bomb survivors supported the LNT model [2]. However, the health effects of LDIR exposure remain to be explored due to the confounding factors related to individual variability, life style, and genetic background [3, 4]. There is increasing concern regarding the health risks arising from LDIR exposure in medical diagnostics and radiation therapy. For example, computed tomography (CT) is a valuable diagnostic imaging technique, but its overuse raises concerns about the potential risks of iatrogenic cancers [5–7]. CT-guided interventional procedures result in greater dose exposure than a routine CT and have a higher risk [8][9].

In regard of the underlying mechanisms of LDIR’s effects on skin structures, it was reported that LDIR promoted IL-12 production, dendritic cells migration [10], and skin-infiltrating monocytes/macrophages migration to draining lymph nodes [11]. Interestingly, ionizing radiation affects not only the directly irradiated cells but also non-irradiated neighboring cells. The phenomenon involving non-irradiated cells is known as the radiation-induced bystander effect (RIBE). Since Nagasawa and Little first reported the RIBE [12], several studies have detected this phenomenon [13–15]. However, little is still known about the effects of LDIR and RIBE on individual cells composing skin structures. In this study, we investigated the molecular effects of LDIR on *in vitro* human primary keratinocytes and U937 cell lines, monocyte-like histiocytic lymphoma cells. Coexistence of these cells may mimic a skin-infiltrating model to assess the contribution of monocytic cells to the release of inflammatory cytokines and the interactions between monocytic cells and irradiated neighboring skin cells.

## Materials and methods

### Cell cultures and reagents

U937 cells were purchased from the American Type Culture Collection (Rockville, MD, USA) and cultured in RPMI 1640 medium containing 1% penicillin-streptomycin with 10% fetal bovine serum. Human primary keratinocytes (HPKs) taken from new born (KK-4009, Kurabo Industries, Osaka, Japan) were cultured in HuMedia-KG2 medium (Kurabo) supplemented with recombinant human epidermal growth factor (0.1 ng/mL), recombinant human insulin (10 μg/mL), hydrocortisone (0.67 μg/mL), gentamicin (50 μg/mL), amphotericin B (50 ng/mL) (Kurabo), and bovine pituitary extract (0.004 mg/mL) which has similar mitogenic activity with fetal bovine serum[16, 17]. HPKs at passage 3 were used for the experiments.

For LDIR exposure, the U937 cells and HPKs were irradiated with 0.1 Gy. All irradiation was performed with 4 MeV X-rays generated by a linear accelerator (Clinac21EX, Varian, Palo Alto, CA, USA) following full build-up (1 cm) at a dose rate of 2.0 Gy/min, as previously reported [18]. Cells were cultured for 24 hours after irradiation. For the bystander experiments, the culture medium of irradiated and sham-irradiated U937 cells and HPKs was extracted 24 hours after irradiation and incubated bystander cells with the extracted medium for another 24 hours and harvested them for gene expression or protein analyses. U937-(IR)-BS means U937 cells after 24 hour incubation with medium from irradiated HPKs, and HPK-(IR)-BS means HPKs after incubation with medium from irradiated U937 cells.

### cDNA microarray

Gene expression in the cells was determined by microarray analysis using the Affymetrix Human Gene 2.0 ST Array, according to the Affymetrix protocols (Santa Clara, CA, USA). Signal intensities were measured using a GeneChip Scanner3000 7G (Affymetrix) and converted to numerical data using the Affymetrix Expression Console software 1.3.1 (Affymetrix). To identify candidate genes of potential significance in U937, we applied a 1.2-fold change cutoff, since the combined responses of a group of genes acting in concert might affect the physiology of the cell, as previously described [19]. The digitized data were analyzed using GeneSpring GX 13.1.0 software (Agilent Technologies, Santa Clara, CA, USA). Genes whose expression changed significantly with treatment were subjected to functional analysis using Ingenuity Pathway Analysis software (IPA, Ingenuity Systems, QIAGEN, www.qiagen.com/ingenuity) [20]. For IPA analyses, we used two scores: an ‘enrichment’ score (Fisher’s exact test *P*-value) that measures the overlap of observed and predicted regulated gene sets, and a Z-score that assesses the match of observed and predicted upregulation/downregulation patterns [21]. The inclusion criteria for genes selected for the analysis was a fold change > ± .8 [22].

### isobaric tags for relative and absolute quantification (iTRAQ) sample labeling, mass spectrometry analysis, and peptide identification

Proteins in treated cells were identified using iTRAQ, a chemical labeling mass spectrometry (MS) method that was performed according to the manufacturer’s protocol (AB SCIEX, Framingham, MA, USA) [23, 24]. Briefly, labeled peptides were analyzed using nano liquid chromatography in combination with tandem mass spectrometry (LC-MS/MS). Nano LC-MS was performed on a nano LC system (AB SCIEX) using a ChromXP C18-CL column (Eksigent, AB SCIEX, Dublin, CA, USA) and a TripleTOF 5600 mass spectrometer for MS/MS (AB SCIEX) with Analyst TF 1.7 software. Proteins were identified and quantified relatively using ProteinPilot ver. 5.0 software (AB SCIEX) [25]. The functions of the identified proteins were determined by searching the UniProt database (released on 01/20/2016). Protein ratios were normalized against the overall median ratio for all of the peptides in the sample for each separate ratio in every individual experiment. A confidence cut-off for protein identification of >95% was applied. The proteins whose expression levels were changed by more than 1.6-fold after irradiation were defined as “profoundly modulated genes” as previously described [19], and the data were submitted to IPA [20].

### Immunoblot analysis

Cells were solubilized in lysis buffer comprising phosphate-buffered saline solution containing 1× cell lysis buffer (Cell Signaling Technology, Danvers, MA, USA), 1× protease inhibitor cocktail (Roche, Indianapolis, IN, USA), and 1× phosphatase inhibitor cocktail (Roche). The mixtures were incubated for 30 min on ice. The lysates were, then, centrifuged for 10 min at a speed of 13,000 rpm at 4°C. Total protein concentrations were determined using the Bio-Rad Protein Assay Kit (Bio-Rad, Hercules, CA, USA) according to the manufacturer’s instructions. Total proteins (20 μg) were separated by sodium dodecyl sulfate-polyacrylamide gel electrophoresis (Bio-Rad) and transferred to polyvinylidene-fluoride membranes (0.45 μm, Millipore, Bedford, MA, USA), and subsequently probed with first and second antibodies. The following antibodies were used: α-tubulin (Sigma-Aldrich, St Louis, MO, USA), p21^WAF1/CIP1^, HIF1α (BD Biosciences, San Jose, CA, USA), c-Myc, PP2Aα, p-4EBP1, 4EBP-1, p-S6 ribosomal protein^Ser235/Ser236^, S6 ribosomal protein, p-p38 MAPK, p38 MAPK, and horseradish peroxidase-linked anti-mouse and anti-rabbit IgG (all from Cell Signaling Technology).

### Statistical analyses

Groups were compared using a two-tailed Student’s *t*-test. A *P*-value ≤0.05 was considered statistically significant. Where indicated, the results are expressed as the mean ± standard deviation (SD) of triplicate samples.

## Results

### LDIR induced gene expression changes in U937 cells

To investigate changes in gene expression caused by LDIR, we performed a cDNA microarray analysis of U937 cells that had been exposed to 0.1 Gy of X-rays in direct or bystander conditions, and then cultured for 24 hours. The cDNA microarray analysis showed that direct LDIR exposure increased expression of the *CARD9*, *HIST1H2BH*, and *mir4497* genes in U937cells (U937-IR) (>1.8-fold change) while no genes showed decreased expression (S1 Table). *CARD9* is a member of the caspase recruitment domain (CARD) family, an upstream activator of BCL10 and NF-κB signaling that plays a regulatory role in cell apoptosis [26]. *HIST1H2BH* has no intron and encodes a member of the histone H2B family and plays roles in DNA repair and replication [27–29]. In U937-bystander [U937-(IR)-BS] cells, 24 genes were downregulated compared with the control cells, including *MT-TG*, *mir 4659*, *RAD51D*, *MT-TR*, *mir 4295*, *MT-TL2*, *mir 644A*, *mir 4521*, and *HIST1H4D* (S1 Table). *MT-TG*, *MT-TR*, and *MT-TL2* are transfer RNAs. *RAD51D*, a member of the RAD51 family, is involved in homologous recombination repair after DNA damage. No upregulated genes were detected in U937-(IR)-BS cells.

We, then, investigated the candidate upstream regulators that can be involved in the responses to LDIR in U937 cells. IPA analysis highlighted the activation of eight upstream factors in U937-IR cells, including TNF, CSF2, and IFNG. In U937-(IR)-BS cells, 10 upstream factors were activated including transforming growth factor beta 1 (TGFβ1) (>1.8-fold change), and 21 were inhibited including hypoxia-inducible factor 1 alpha (HIF1α) (Table 1). The upregulation of TGFβ was concordant with the previous reports by us [30] and the others [31, 32].

**Table 1:** Upstream factors involved in alterations of gene transcription caused by LDR in U937 cells (at 24 hours).

Upstream Regulator	Activation z-score	P-value
U937-IR		
Activated		
TNF	2.18	3.11E-01
CSF2	2.13	2.31E-01
IFNG	2.07	2.14E-01
E2F3	1.98	2.29E-02
Akt	1.97	8.86E-02
IL5	1.96	1.49E-01
IL1B	1.96	1.00E00
IL1A	1.95	7.62E-02
Inhibited		
N.A.		
U937-(IR)-BS		
Activated		
MGEA5	2.84	5.13E-01
FSH	2.08	1.00E00
SFTPA1	2.00	3.51E-01
Nr1h	1.98	1.00E00
SYVN1	1.98	3.32E-03
MMP3	1.90	1.01E-02
FLT1	1.89	2.62E-02
AKT1	1.83	3.59E-01
TGFB1	1.82	1.00E00
KLF4	1.81	1.00E00
Inhibited		
REL	-2.58	1.39E-01
SMARCA4	-2.56	4.78E-01
IRF7	-2.42	6.75E-02
HIF1A	-2.41	5.34E-01
TNF	-2.23	1.00E00
TICAM1	-2.22	1.00E00
TNFRSF1A	-2.22	1.00E00
Ifn	-2.19	5.22E-01
NR1H4	-2.18	1.00E00
S100A9	-2.16	3.22E-01
PKD1	-2.00	1.00E00
HNF4A	-1.99	1.01E-03
IKBKG	-1.98	5.52E-01
CXCL12	-1.94	1.31E-01
CSF2	-1.93	1.00E-01
S100A8	-1.92	3.89E-01
IRF5	-1.89	2.13E-02
CST5	-1.89	3.34E-02
CXCR4	-1.88	7.55E-02
FOXO1	-1.82	2.11E-01
IFNG	-1.82	1.00E00

Data were analyzed using Ingenuity Pathway Analysis (IPA) based on genes for which transcription was consistently altered after treatment in U937 cells.

To exclude the changes caused by the mix and match protocols in the medium transfer experiments, we confirmed that fully supplemented RPMI 1640 medium and HuMedia-KG2 medium caused no significant difference of cell viability after the LDIR directly or in the bystander condition by the trypan blue exclusion cell count method (data not shown).

### LDIR activated the cytokines associated with inflammatory pathways in U937 cells

We next investigated the changes in protein expression and molecular networks with LDIR in direct and bystander conditions of U937 cells using iTRAQ proteomic approach.

**Fig 1:**
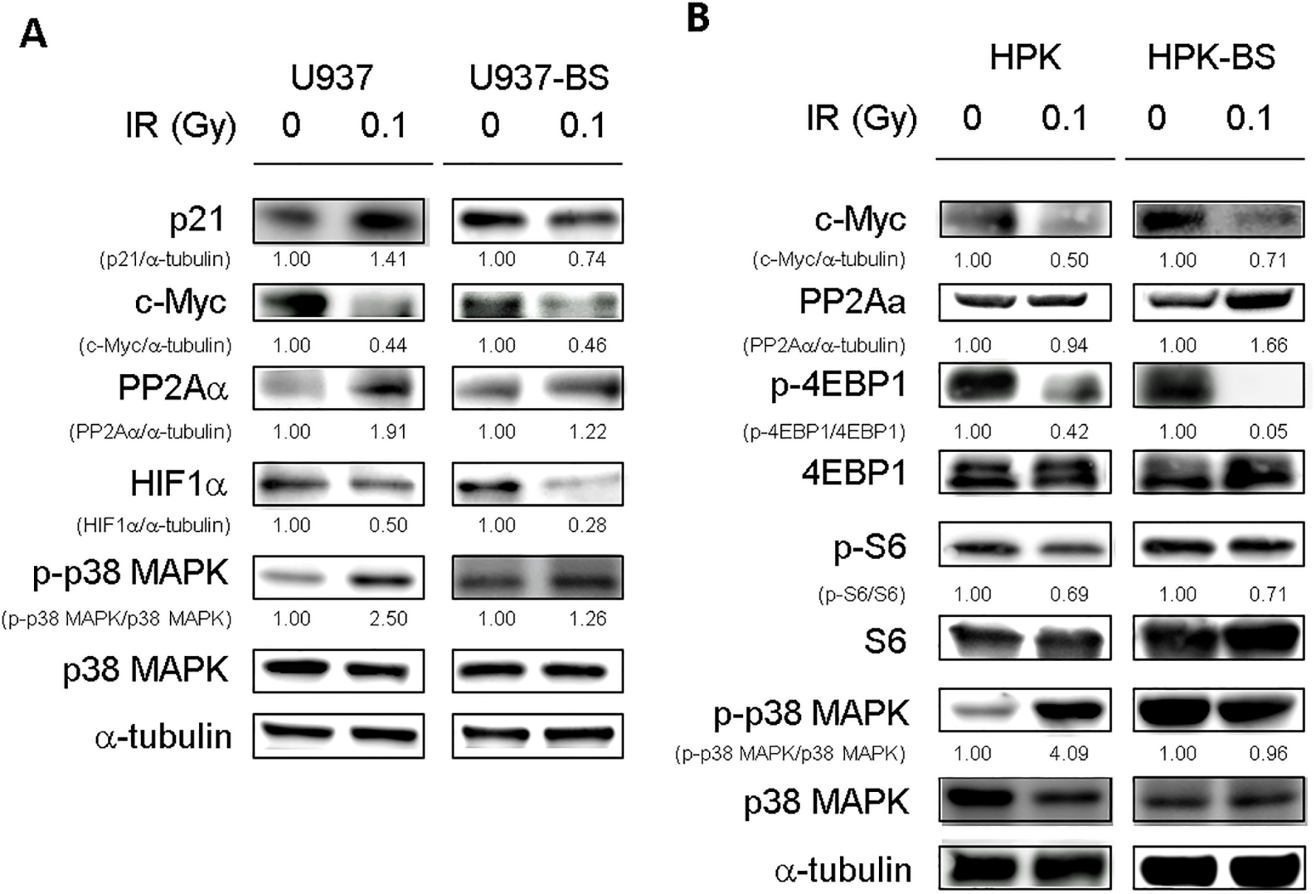
Molecular pathways affected by LDIR in U937 cells and HPKs. After direct LDIR for 24 hours or in the bystander condition, the cells were subjected to lysis and immunoblot analyses. The results are representative of three independent experiments, and the intensity of each immunoblot signal compared with that of α-tubulin was quantified using ImageJ software; the quantity is shown directly under each blot.

In U937-IR, 2276 unique proteins were identified including 61 proteins (28 upregulated and 33 downregulated) whose expression levels were significantly altered by the LDIR treatment. In U937-(IR)-BS cells, 1385 proteins were identified with 32 significantly altered proteins (13 upregulated, 19 downregulated) (S2 Table). We, then, examined the candidate upstream regulators and network interactions involved in the responses to LDIR in U937 cells. As shown in Table 2, IPA analyses highlighted the activation of the proliferation and differentiation modulator oncostatin M (OSM) in U937-IR and the inhibition of HIF1α in U937-(IR)-BS cells. OSM is a member of the IL-6 family of cytokines that is associated with skin inflammation [33]. The network analyses further demonstrated the activation of protein phosphatase 2A (PP2A), a negative cell growth regulator, in U937-IR cells (S1A Fig). The immunoblot analyses confirmed the observation that LDIR increased PP2Aα in both U937-IR and U937-(IR)-BS cells, which is consistent with the iTRAQ results (Fig 1A). PP2A is a family of serine/threonine phosphatases that negatively regulates mTOR signaling [34–36]. Because c-Myc is controlled by PP2A [37] and our cDNA microarray results indicated the LDR-induced activation of TGFβ signaling, another negative regulator of c-Myc [38, 39], we investigated the changes in the expression of c-Myc. As shown in Fig 1A, immunoblot analyses demonstrated that LDIR suppressed c-Myc protein levels in both U937-IR and U937-(IR)-BS cells. We also detected the downregulation of p21^WAF1/CIP1^, a negative downstream target of c-Myc[40, 41] that arrests cell cycle/growth in U937-IR cells. In addition, p38 MAPK, a downstream factor of TGF-β [42–44], was activated in both U937 and U937-BS cells after irradiation. Immunoblotting analyses confirmed the cDNA array findings (*i*.*e*., the downregulation of HIF1α in both U937-IR and U937-(IR)-BS cells (Fig 1A)). These results suggest that LDR plays a role in changing the various cytokine pathways via the activation of TGFβ and inactivation of OSM.

**Table 2:** Upstream factors involved in the protein expression responses to LDIR in U937 cells and HPKs (at 24 hours).

Upstream Regulator	Activation z-score	p-value of overlap	Target molecules in dataset
U937-IR			
Activated			
OSM	1.63	4.15E-03	ARHGEF2,GART,LRRFIP1,OGDH,PCNA,PGK1
Inhibited			
PRL	-2.39	1.32E-04	CTSD,MSN,P4HB,PCNA,RPSA,YWHAG
EGFR	-1.67	1.42E-05	CCT5,HNRNPA2B1,HSPE1,IGBP1,PCNA,PPIA,PSMB1,TUBA4A
U937-(IR)-BS			
Activated			
TP53	1.84	6.61E-03	ACTN1,ARPC1B,EZR,HSPB1,PCNA,SFPQ,SOD1
Inhibited			
HIF1A	-2.21	2.42E-05	ALDOA,EIF5A,HSPA5,HSPB1,PGK1,PPIA
HPK-IR			
Activated			
FGF2	2.37	5.67E-05	ALB, FLNA, ITGB1, PRKDC, THBS1, XRCC5, XRCC6
IL4	2.24	5.94E-03	ANXA2, IDE, ITGB1, NCL, PRKDC, XRCC5, XRCC6
INSR	2.21	8.61E-05	ACTN4, ATP5B, FH, HADHA, HSPD1, MDH2, RAB7A
IFNG	2.01	6.05E-02	HSPD1, IDE, ITGB1, KRT14, LAMC2, PSME3, THBS1
ESRRA	1.95	1.34E-03	ATP5B, HADHA, HK1, LDHA
TNF	1.90	1.90E-02	ALB,HSPD1,IDE,ITGB1,LAD1,LAMC2,LDHA,RPS3,THBS1,TPP2
TP63	1.68	3.83E-03	CAD,ITGB1,KRT14,KRT6A,THBS1
PRL	1.62	9.94E-05	ACTR3,ANXA2,HSPD1,IDE,KRT14,KRT5
Inhibited			
CST5	-2.00	6.95E-03	AHNAK,ANXA2,HNRNPU,NCL
CD3	-1.67	1.21E-07	ACTN4,ACTR3,HNRNPA1,KRT14,MDH2,NCL,NPM1,SF1,SSB,THBS1,XRCC6,YARS
CD28	-1.63	3.52E-04	ACTR3,HNRNPA1,NCL,SSB,THBS1,XRCC6

Data were analyzed by Ingenuity Pathway Analysis (IPA) based on the proteins whose expression was consistently altered after the indicated treatment in U937 cells and HPKs (S2 Table).

### Proteome analysis detected ribosomal biogenesis inhibition by LDIR in HPKs

In HPK-IR cells, 1551 unique proteins were identified including 58 proteins (22 upregulated and36 downregulated) whose expression levels were significantly altered by LDIR treatment (S1 Table). In HPK-(IR)-BS cells, 1658 proteins were identified with 21 significantly altered proteins (3 upregulated and, 18 downregulated) proteins whose expression levels were significantly altered by LDIR treatment (S1 Table). The candidate upstream regulators and network interactions involved in the responses to LDIR in HPKs were examined by IPA, which highlighted the activation of the anti-inflammatory cytokine interleukin-4 (IL-4), tumor necrosis factor (TNF), and the tumor suppressor protein TP63 in LDIR -irradiated HPKs (HPK-IR) (Table 2). IL-4 promotes the activation of macrophages as M2 cells, which reduces inflammation [45]. TNF is a cytokine that can induce apoptosis and inflammation. TP63 is a member of the TP53 family. The network analyses further demonstrated the activation of heat shock protein 70 (HSP70) in HPK-IR cells (S1C Fig) and the downregulation of heat shock protein 90 (HSP90) and the proinflammatory cytokine high mobility group box 1 (HMGB1) [46–48] in HPK-(IR)-BS cells (S1D Fig). HSP70 and HSP90 are molecular chaperones that protect cells against damage to the proteome and assist in the refolding of denatured proteins and regulating degradation after severe protein damage [49] via mutual interaction [50]. Because IPA analyses indicated that LDIR irradiation upregulated TNF, we examined the activation of its downstream factor p38 MAPK [51], and found that LDIR irradiation increased the expression of phospho-p38 MAPK in HPK-IR cells (Fig 1B). Since IPA analyses also indicated that LDIR irradiation repressed HSP90, the synthesis of which is regulated by mTOR [52], we further investigated the changes in mTOR signaling. We observed that LDIR irradiation suppressed the mTOR signaling along with decreased phospho-S6 and phospho-4EBP1 protein expression in both HPK-IR and HPK-(IR)-BS cells (Fig 1B). In addition, the expression of PP2Aα, an upstream molecule of mTOR[34] and c-Myc controlled by PP2Aα [37] were investigated. The immunoblotting demonstrated that LDIR increased the expression of PP2A in HPK-(IR)-BS cells and decreased the expression of c-Myc in HPK-IR and HPK-(IR)-BS cells (Fig 1B). PP2A inhibits c-Myc and mTOR signaling, including S6 and 4EBP1. Because mTOR signaling controls protein synthesis by inducing ribosome biogenesis and translation [53–55], these results indicate that LDIR irradiation suppresses ribosomal biogenesis via the upregulation of PP2Aα and downregulation of c-Myc and mTOR signaling.

## Discussion

This study demonstrated several findings: (1) the U937 cells, mimicking skin-infiltrating monocytes, released inflammatory cytokines after LDIR; (2) the interactions between U937 cells and irradiated neighboring keratinocytes in LDIR exposure; and (3) LDIR also inhibited the ribosomal biogenesis in keratinocytes and U937 cells in *in vitro*.

After LDIR exposure, communication with sham-irradiated bystander cells via bioactive substances from irradiated cells modifies biological responses, and the RIBE can become saturated at relatively low doses of irradiation [56]. Furthermore, the RIBE of LDIR is as effective as direct irradiation [56–58]. It is known that TGFβ inhibits cell growth as a direct effect of irradiation as well as RIBE mediators [59, 60]. In this study, we demonstrated that direct LDIR irradiation and RIBE downregulated c-Myc, a negative downstream factor of TGFβ, both in U937 cells and HPKs. TGFβ and p21^WAF1/CIP1^, an activator of TGFβ, were upregulated in bystander U937 cells. Moreover, TNF-α is another important mediator of RIBE [61]. We detected that LDIR activated TNFα and p38 MAPK, which plays an essential role in RIBE [43, 62] via TNFα [63]. As a result, LDIR released inflammatory cytokines including TNFα, induced cell growth arrest via TGFβ/c-Myc/p21 ^WAF1/CIP^ pathways and caused RIBE through the activation of TGFβ and TNFα.

In addition to the above observations, we first found that LDIR increased PP2A, which negatively regulates the cell growth and inhibits c-Myc [64]. The downregulated c-Myc might upregulate p21^WAF1/CIP1^ proteins. These facts suggest that LDIR might arrest cell growth via the PP2A/c-Myc/p21^WAF1/CIP^ pathways. We also detected that LDIR repressed S6 and 4EBP1 activation, as mTOR targets. It has been known that repression of mTOR signaling, in which PP2A possibly plays a role in inhibiting ribosomal biogenesis [65–67]. Therefore, LDIR could repress ribosomal biogenesis via PP2A/mTOR signaling pathway. We further observed that LDIR upregulated the OSM which is known to be associated with cell growth inhibition [68].

**Fig 2:**
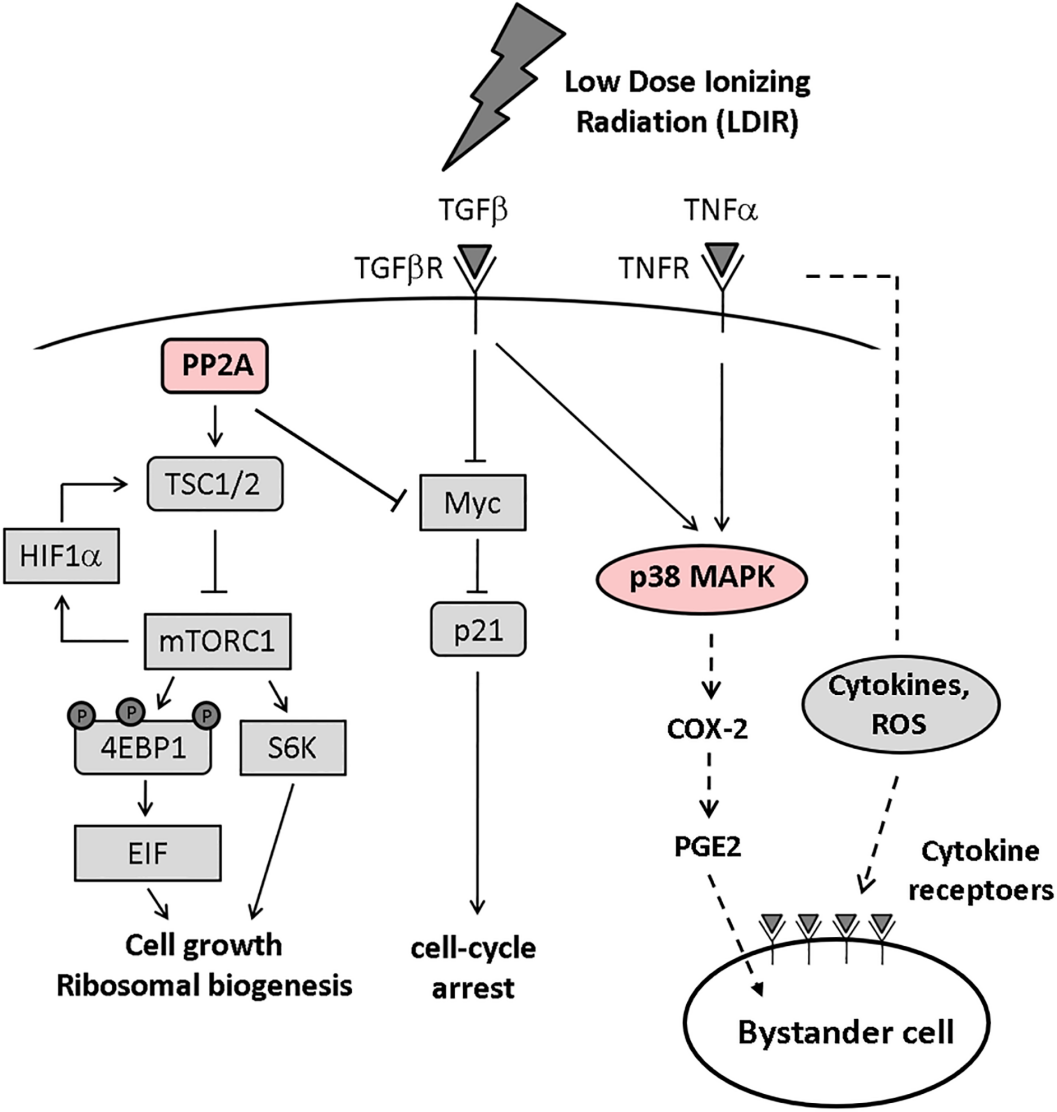
LDIR inhibits cell growth and protein synthesis and induces bystander effects. Cell signaling pathways affected by LDIR. LDIR downregulates c-Myc and upregulates p21 ^WAF1/CIP1^ via stimulation of TGFβ and PP2A. PP2A also inhibits mTOR signaling with repression of S6K activation and 4EBP1 phosphorylation that resulted in decrease in protein synthesis. Furthermore, LDIR induces bystander effects through p38 MAPK activation. Directly-irradiated cells release cytokine signals that affect non-irradiated (bystander) cells.

As the study limitations, this study investigated gene and protein expression at single time point using only two types of cell lines in *in vitro* culture. This study focused on the changes after 24 hours based on the previous reports that demonstrated the bystander effects on molecular pathways changed after 16–24 hours [69, 70]. Many studies have shown that gene and/or protein expression patterns are dependent on time after irradiation [30, 69, 71]. On the other hand, we previously demonstrated whereas proteomics data showed little overlapping responses observed at two post-radiation time points (i.e. 1 hour and 24 hours), the pathway analysis demonstrated the connections between the time points depending on LDIR conditions [30]. The further experiments at earlier or later time points are required to elucidate how much the molecular pathway changes dependent on time after irradiation. And *in vivo* studies are warranted to validate our current observation and evaluate the LDIR effects. In addition, we used keratinocytes and monocytic cell lines (U937 cells) as the skin-infiltrating model in inflammation induced by LDIR which is different from actual human skin structures. Taken together, our findings indicate that LDIR affects cell cycle and protein synthesis pathways with activation of PP2A and p38 MAPK, in skin-infiltrating monocytic cells directly and indirectly via interactions with irradiated neighboring normal skin cells via bystander effects (Fig 2). LDIR exposure, including iatrogenic exposure with diagnostic imaging, may cause molecular alterations associating with skin inflammation and proliferation in our skin-infiltrating model.

## Supporting information

S1 Table Genes with altered expression in U937 cells after 0.1 Gy X-ray irradiation. Genes that showed a fold-change >1.7 compared with controls are shown.
(DOCX)

S2 Table Proteins with altered expression in U937 cells and HPKs after 0.1 Gy X-ray irradiation. The protein expression levels in U937 cells and HPKs were detected by two independent iTRAQ experiments. The protein expression changes measured three times in each experiment. The expression of all proteins listed differed significantly (*P* < 0.05) between controls and cells exposed to LDIR. Values indicate the fold-change relative to untreated cells. The confidence score (a percentage measure of the confidence of protein identification) for all proteins in the table was 99%.
(DOCX)

S1 Fig Network of proteins involved in the responses of U937 cells and HPKs to LDIR. Data were analyzed using IPA (QIAGEN, www.qiagen.com/ingenuity). The IPA network analysis showed direct interactions between differentially expressed molecules in U937 cells and HPKs after the LDIR treatment directly or in the bystander condition. U937-IR cells; (B) U937-(IR)-BS cells; (C) HPK-IR cells; (D) HPK-(IR)-BS cells. Arrows indicate direct interactions between molecules. Lines represent direct (solid lines) and indirect (dashed lines) interactions between molecules. The network with the highest score is shown. Upregulated proteins in the dataset are depicted in pink and downregulated proteins in green. The depth of color indicates the degree of change [72].
(TIF)
