# Correction: Low-dose ionizing radiation exposure represses the cell cycle and protein synthesis pathways in *in vitro* human primary keratinocytes and U937 cell lines

**DOI:** 10.1371/journal.pone.0205581

**Published:** 2018-10-05

**Authors:** Kazumasa Sekihara, Kaori Saitoh, Haeun Yang, Haruki Kawashima, Saiko Kazuno, Mika Kikkawa, Hajime Arai, Takashi Miida, Nobuhiro Hayashi, Francois Niyonsaba, Keisuke Sasai, Yoko Tabe

Dr. Francois Niyonsaba is not included in the author byline. He should be listed as the tenth author, and his affiliation is the Atopy Research Center, Juntendo University Graduate School of Medicine, Tokyo, Japan. The contributions of this author are as follows: Conceptualization and resources.

The following information is missing from the Funding section: This study was supported by Grant-in-Aid for Young Scientists (B) (16K19855) from the MEXT, Japan to KS.
