# Supplementary material for: Low-dose ionizing radiation exposure represses the cell cycle and protein synthesis pathways in in vitro human primary keratinocytes and U937 cell lines
Source: PLoS One. 2018 Jun 18;13(6):e0199117. doi: 10.1371/journal.pone.0199117 (PMC6005503; doi:10.1371/journal.pone.0199117)
Supplement: S1 Table — Genes that showed a fold-change >1.7 compared with controls are shown. (DOCX) [file pone.0199117.s001.docx]

**S1 Table**. **Genes with altered expression in U937 cells after 0**.**1 Gy X-ray irradiation**.

| Gene Symbol | Fold Change |
| --- | --- |
| U937-IR |  |
| Upregulated |  |
| CARD9 | 1.73 |
| HIST1H2BH | 1.72 |
| mir4497 | 1.72 |
| Downregulated |  |
| N.A. |  |
| U937-(IR)-BS |  |
| Upregulated |  |
| N.A. |  |
| Downregulated |  |
| MT-TG | -3.55 |
| mir 4659A\| mir 4659B | -2.79 |
| RAD51D | -2.77 |
| MT-TR | -2.61 |
| mir 4295 | -2.37 |
| MT-TL2 | -2.16 |
| mir 644A | -2.00 |
| mir 4521 | -2.00 |
| HIST1H4D | -1.96 |
| MT-TM | -1.93 |
| CCDC71 | -1.89 |
| HIST1H3B | -1.89 |
| mir 548I3 | -1.85 |
| AFF2-IT1 | -1.84 |
| TAS2R30 | -1.82 |
| MT-TQ | -1.81 |
| GNG5P2 | -1.80 |
| MRPL42P5 | -1.79 |
| PTGR2\|RP5-1021I20.4 | -1.76 |
| HIST1H2BB | -1.76 |
| mir 548I2\| mir 548I1\| mir 548I3 | -1.75 |
| HIST1H1B | -1.74 |
| mir4454 | -1.73 |
| MNS1 | -1.71 |
