# Supplementary material for: Low-dose ionizing radiation exposure represses the cell cycle and protein synthesis pathways in in vitro human primary keratinocytes and U937 cell lines
Source: PLoS One. 2018 Jun 18;13(6):e0199117. doi: 10.1371/journal.pone.0199117 (PMC6005503; doi:10.1371/journal.pone.0199117)
Supplement: S2 Table — The protein expression levels in U937 cells and HPKs were detected by two independent iTRAQ experiments. The protein expression changes measured three times in each experiment. The expression of all proteins listed differed significantly (P < 0.05) between controls and cells exposed to LDIR. Values indicate the fold-change relative to untreated cells. The confidence score (a percentage measure of the confidence of protein identification) for all proteins in the table was 99%. (DOCX) [file pone.0199117.s002.docx]

**S2 Table**. **Proteins with altered expression in U937 cells and HPKs after 0**.**1 Gy X-ray irradiation**.

| Gene Symbol | Protein Name | Fold Change | | |
| --- | --- | --- | --- | --- |
| U937-IR |  |  |  |  |
| Upregulated |  |  |  |  |
| NUP107 | Nuclear pore complex protein Nup107 OS=Homo sapiens GN=NUP107 PE=1 SV=1 | 4.16 | ± | 0.04 |
| S100A11 | Protein S100-A11 OS=Homo sapiens GN=S100A11 PE=1 SV=2 | 1.74 | ± | 0.02 |
| IGBP1 | Immunoglobulin-binding protein 1 OS=Homo sapiens GN=IGBP1 PE=1 SV=1 | 1.52 | ± | 0.01 |
| ZRANB2 | Isoform 2 of Zinc finger Ran-binding domain-containing protein 2 OS=Homo sapiens GN=ZRANB2 | 1.43 | ± | 0.03 |
| SSR1 | Translocon-associated protein subunit alpha OS=Homo sapiens GN=SSR1 PE=1 SV=3 | 1.40 | ± | 0.02 |
| DBI | Acyl-CoA-binding protein OS=Homo sapiens GN=DBI PE=1 SV=2 | 1.37 | ± | 0.00 |
| ATP5L | ATP synthase subunit g, mitochondrial OS=Homo sapiens GN=ATP5L PE=1 SV=3 | 1.36 | ± | 0.05 |
| PPP2R5D | Serine/threonine-protein phosphatase 2A 56 kDa regulatory subunit delta isoform OS=Homo sapiens GN=PPP2R5D PE=1 SV=1 | 1.34 | ± | 0.04 |
| CLIC1 | Chloride intracellular channel protein 1 OS=Homo sapiens GN=CLIC1 PE=1 SV=4 | 1.34 | ± | 0.00 |
| SASH3 | SAM and SH3 domain-containing protein 3 OS=Homo sapiens GN=SASH3 PE=1 SV=2 | 1.34 | ± | 0.03 |
| OGFR | Opioid growth factor receptor OS=Homo sapiens GN=OGFR PE=1 SV=3 | 1.31 | ± | 0.04 |
| UBE2K | Ubiquitin-conjugating enzyme E2 K OS=Homo sapiens GN=UBE2K PE=1 SV=3 | 1.29 | ± | 0.02 |
| ANPEP | Aminopeptidase N OS=Homo sapiens GN=ANPEP PE=1 SV=4 | 1.28 | ± | 0.03 |
| DCXR | L-xylulose reductase OS=Homo sapiens GN=DCXR PE=1 SV=2 | 1.26 | ± | 0.05 |
| M6PR | Cation-dependent mannose-6-phosphate receptor OS=Homo sapiens GN=M6PR PE=1 SV=1 | 1.21 | ± | 0.04 |
| FBL | rRNA 2'-O-methyltransferase fibrillarin OS=Homo sapiens GN=FBL PE=1 SV=2 | 1.21 | ± | 0.03 |
| KPNA2 | Importin subunit alpha-1 OS=Homo sapiens GN=KPNA2 PE=1 SV=1 | 1.20 | ± | 0.04 |
| PSMB1 | Proteasome subunit beta type-1 OS=Homo sapiens GN=PSMB1 PE=1 SV=2 | 1.20 | ± | 0.02 |
| IPO5 | Importin-5 OS=Homo sapiens GN=IPO5 PE=1 SV=4 | 1.19 | ± | 0.02 |
| LRRFIP1 | Isoform 3 of Leucine-rich repeat flightless-interacting protein 1 OS=Homo sapiens GN=LRRFIP1 | 1.16 | ± | 0.01 |
| P4HB | Protein disulfide-isomerase OS=Homo sapiens GN=P4HB PE=1 SV=3 | 1.16 | ± | 0.01 |
| PDCD5 | Programmed cell death protein 5 OS=Homo sapiens GN=PDCD5 PE=1 SV=3 | 1.16 | ± | 0.04 |
| ARHGEF2 | Rho guanine nucleotide exchange factor 2 OS=Homo sapiens GN=ARHGEF2 PE=1 SV=4 | 1.14 | ± | 0.01 |
| PSMD13 | 26S proteasome non-ATPase regulatory subunit 13 OS=Homo sapiens GN=PSMD13 PE=1 SV=2 | 1.12 | ± | 0.03 |
| CSDE1 | Cold shock domain-containing protein E1 OS=Homo sapiens GN=CSDE1 PE=1 SV=2 | 1.10 | ± | 0.05 |
| MCM6 | DNA replication licensing factor MCM6 OS=Homo sapiens GN=MCM6 PE=1 SV=1 | 1.08 | ± | 0.03 |
| GART | Trifunctional purine biosynthetic protein adenosine-3 OS=Homo sapiens GN=GART PE=1 SV=1 | 1.08 | ± | 0.04 |
| AHNAK | Neuroblast differentiation-associated protein AHNAK OS=Homo sapiens GN=AHNAK PE=1 SV=2 | 1.08 | ± | 0.02 |
|  |  |  |  |  |
| Downregulated |  |  |  |  |
| PPIA | Peptidyl-prolyl cis-trans isomerase A OS=Homo sapiens GN=PPIA PE=1 SV=2 | 0.62 | ± | 0.00 |
| UBE2I | SUMO-conjugating enzyme UBC9 OS=Homo sapiens GN=UBE2I PE=1 SV=1 | 0.67 | ± | 0.04 |
| FTL | Ferritin light chain OS=Homo sapiens GN=FTL PE=1 SV=2 | 0.68 | ± | 0.02 |
| RPSA | 40S ribosomal protein SA OS=Homo sapiens GN=RPSA PE=1 SV=4 | 0.71 | ± | 0.00 |
| LBR | Lamin-B receptor OS=Homo sapiens GN=LBR PE=1 SV=2 | 0.74 | ± | 0.01 |
| CFL1 | Cofilin-1 OS=Homo sapiens GN=CFL1 PE=1 SV=3 | 0.75 | ± | 0.00 |
| PLEK | Pleckstrin OS=Homo sapiens GN=PLEK PE=1 SV=3 | 0.77 | ± | 0.00 |
| CRKL | Crk-like protein OS=Homo sapiens GN=CRKL PE=1 SV=1 | 0.78 | ± | 0.04 |
| PEBP1 | Phosphatidylethanolamine-binding protein 1 OS=Homo sapiens GN=PEBP1 PE=1 SV=3 | 0.78 | ± | 0.03 |
| HSPE1 | 10 kDa heat shock protein, mitochondrial OS=Homo sapiens GN=HSPE1 PE=1 SV=2 | 0.79 | ± | 0.04 |
| TUBA4A | Tubulin alpha-4A chain OS=Homo sapiens GN=TUBA4A PE=1 SV=1 | 0.79 | ± | 0.05 |
| HNRNPA2B1 | Heterogeneous nuclear ribonucleoproteins A2/B1 OS=Homo sapiens GN=HNRNPA2B1 PE=1 SV=2 | 0.79 | ± | 0.01 |
| TUBB | Tubulin beta chain OS=Homo sapiens GN=TUBB PE=1 SV=2 | 0.79 | ± | 0.04 |
| APEX1 | DNA-(apurinic or apyrimidinic site) lyase OS=Homo sapiens GN=APEX1 PE=1 SV=2 | 0.80 | ± | 0.04 |
| OGDH | 2-oxoglutarate dehydrogenase, mitochondrial OS=Homo sapiens GN=OGDH PE=1 SV=3 | 0.80 | ± | 0.03 |
| UBXN1 | UBX domain-containing protein 1 OS=Homo sapiens GN=UBXN1 PE=1 SV=2 | 0.81 | ± | 0.05 |
| RPS27A | Ubiquitin-40S ribosomal protein S27a OS=Homo sapiens GN=RPS27A PE=1 SV=2 | 0.82 | ± | 0.01 |
| MAT2B | Isoform 2 of Methionine adenosyltransferase 2 subunit beta OS=Homo sapiens GN=MAT2B | 0.83 | ± | 0.04 |
| PGK1 | Phosphoglycerate kinase 1 OS=Homo sapiens GN=PGK1 PE=1 SV=3 | 0.83 | ± | 0.01 |
| TYMS | Thymidylate synthase OS=Homo sapiens GN=TYMS PE=1 SV=3 | 0.84 | ± | 0.04 |
| MDH2 | Malate dehydrogenase, mitochondrial OS=Homo sapiens GN=MDH2 PE=1 SV=3 | 0.84 | ± | 0.02 |
| UBA2 | SUMO-activating enzyme subunit 2 OS=Homo sapiens GN=UBA2 PE=1 SV=2 | 0.84 | ± | 0.01 |
| ALDH9A1 | 4-trimethylaminobutyraldehyde dehydrogenase OS=Homo sapiens GN=ALDH9A1 PE=1 SV=3 | 0.86 | ± | 0.01 |
| CCT5 | T-complex protein 1 subunit epsilon OS=Homo sapiens GN=CCT5 PE=1 SV=1 | 0.87 | ± | 0.01 |
| YWHAG | 14-3-3 protein gamma OS=Homo sapiens GN=YWHAG PE=1 SV=2 | 0.87 | ± | 0.05 |
| CTSD | Cathepsin D OS=Homo sapiens GN=CTSD PE=1 SV=1 | 0.87 | ± | 0.02 |
| PCNA | Proliferating cell nuclear antigen OS=Homo sapiens GN=PCNA PE=1 SV=1 | 0.87 | ± | 0.01 |
| DDX5 | Probable ATP-dependent RNA helicase DDX5 OS=Homo sapiens GN=DDX5 PE=1 SV=1 | 0.88 | ± | 0.03 |
| CANX | Calnexin OS=Homo sapiens GN=CANX PE=1 SV=2 | 0.88 | ± | 0.05 |
| HNRNPA3 | Heterogeneous nuclear ribonucleoprotein A3 OS=Homo sapiens GN=HNRNPA3 PE=1 SV=2 | 0.88 | ± | 0.04 |
| RPS7 | 40S ribosomal protein S7 OS=Homo sapiens GN=RPS7 PE=1 SV=1 | 0.89 | ± | 0.05 |
| MSN | Moesin OS=Homo sapiens GN=MSN PE=1 SV=3 | 0.91 | ± | 0.02 |
| CACYBP | Calcyclin-binding protein OS=Homo sapiens GN=CACYBP PE=1 SV=2 | 0.91 | ± | 0.02 |
|  |  |  |  |  |
| U937-(IR)-BS |  |  |  |  |
| Upregulated |  |  |  |  |
| UCK2 | Uridine-cytidine kinase 2 OS=Homo sapiens GN=UCK2 PE=1 SV=1 | 1.83 | ± | 0.03 |
| SPTBN1 | Spectrin beta chain, non-erythrocytic 1 OS=Homo sapiens GN=SPTBN1 PE=1 SV=2 | 1.38 | ± | 0.00 |
| DYNC1I2 | Cytoplasmic dynein 1 intermediate chain 2 OS=Homo sapiens GN=DYNC1I2 PE=1 SV=3 | 1.37 | ± | 0.04 |
| SOD1 | Superoxide dismutase [Cu-Zn] OS=Homo sapiens GN=SOD1 PE=1 SV=2 | 1.32 | ± | 0.03 |
| PTMA | Prothymosin alpha OS=Homo sapiens GN=PTMA PE=1 SV=2 | 1.23 | ± | 0.04 |
| SMC1A | Structural maintenance of chromosomes protein 1A OS=Homo sapiens GN=SMC1A PE=1 SV=2 | 1.18 | ± | 0.01 |
| EZR | Ezrin OS=Homo sapiens GN=EZR PE=1 SV=4 | 1.18 | ± | 0.01 |
| DYNC1H1 | Cytoplasmic dynein 1 heavy chain 1 OS=Homo sapiens GN=DYNC1H1 PE=1 SV=5 | 1.18 | ± | 0.03 |
| LARS | Leucine--tRNA ligase, cytoplasmic OS=Homo sapiens GN=LARS PE=1 SV=2 | 1.18 | ± | 0.03 |
| SFPQ | Splicing factor, proline- and glutamine-rich OS=Homo sapiens GN=SFPQ PE=1 SV=2 | 1.16 | ± | 0.03 |
| HSPB1 | Heat shock protein beta-1 OS=Homo sapiens GN=HSPB1 PE=1 SV=2 | 1.13 | ± | 0.02 |
| NCL | Nucleolin OS=Homo sapiens GN=NCL PE=1 SV=3 | 1.12 | ± | 0.03 |
| RPL6 | 60S ribosomal protein L6 OS=Homo sapiens GN=RPL6 PE=1 SV=3 | 1.12 | ± | 0.05 |
|  |  |  |  |  |
| Downregulated |  |  |  |  |
| RPS12 | 40S ribosomal protein S12 OS=Homo sapiens GN=RPS12 PE=1 SV=3 | 0.57 | ± | 0.02 |
| IMMT | MICOS complex subunit MIC60 OS=Homo sapiens GN=IMMT PE=1 SV=1 | 0.67 | ± | 0.01 |
| PPIB | Peptidyl-prolyl cis-trans isomerase B OS=Homo sapiens GN=PPIB PE=1 SV=2 | 0.67 | ± | 0.02 |
| ACTN1 | Alpha-actinin-1 OS=Homo sapiens GN=ACTN1 PE=1 SV=2 | 0.67 | ± | 0.01 |
| RBM39 | RNA-binding protein 39 OS=Homo sapiens GN=RBM39 PE=1 SV=2 | 0.72 | ± | 0.02 |
| PCMT1 | Protein-L-isoaspartate(D-aspartate) O-methyltransferase OS=Homo sapiens GN=PCMT1 PE=1 SV=4 | 0.74 | ± | 0.02 |
| ARPC1B | Actin-related protein 2/3 complex subunit 1B OS=Homo sapiens GN=ARPC1B PE=1 SV=3 | 0.74 | ± | 0.04 |
| PPIA | Peptidyl-prolyl cis-trans isomerase A OS=Homo sapiens GN=PPIA PE=1 SV=2 | 0.75 | ± | 0.03 |
| ATG3 | Ubiquitin-like-conjugating enzyme ATG3 OS=Homo sapiens GN=ATG3 PE=1 SV=1 | 0.76 | ± | 0.02 |
| YWHAE | 14-3-3 protein epsilon OS=Homo sapiens GN=YWHAE PE=1 SV=1 | 0.76 | ± | 0.01 |
| RAB10 | Ras-related protein Rab-10 OS=Homo sapiens GN=RAB10 PE=1 SV=1 | 0.78 | ± | 0.04 |
| ALDOA | Fructose-bisphosphate aldolase A OS=Homo sapiens GN=ALDOA PE=1 SV=2 | 0.78 | ± | 0.00 |
| CBX3 | Chromobox protein homolog 3 OS=Homo sapiens GN=CBX3 PE=1 SV=4 | 0.78 | ± | 0.03 |
| TWF2 | Twinfilin-2 OS=Homo sapiens GN=TWF2 PE=1 SV=2 | 0.79 | ± | 0.04 |
| EIF5A | Isoform 2 of Eukaryotic translation initiation factor 5A-1 OS=Homo sapiens GN=EIF5A | 0.79 | ± | 0.03 |
| PCNA | Proliferating cell nuclear antigen OS=Homo sapiens GN=PCNA PE=1 SV=1 | 0.81 | ± | 0.05 |
| TALDO1 | Transaldolase OS=Homo sapiens GN=TALDO1 PE=1 SV=2 | 0.83 | ± | 0.03 |
| PGK1 | Phosphoglycerate kinase 1 OS=Homo sapiens GN=PGK1 PE=1 SV=3 | 0.85 | ± | 0.01 |
| HSPA5 | 78 kDa glucose-regulated protein OS=Homo sapiens GN=HSPA5 PE=1 SV=2 | 0.88 | ± | 0.05 |
|  |  |  |  |  |
| HPK-IR |  |  |  |  |
| Upregulated |  |  |  |  |
| KRT9 | Keratin, type I cytoskeletal 9 OS=Homo sapiens GN=KRT9 PE=1 SV=3 | 1.79 | ± | 0.03 |
| KRT1 | Keratin, type II cytoskeletal 1 OS=Homo sapiens GN=KRT1 PE=1 SV=6 | 1.59 | ± | 0.00 |
| RPL21 | 60S ribosomal protein L21 OS=Homo sapiens GN=RPL21 PE=1 SV=2 | 1.44 | ± | 0.02 |
| NONO | Isoform 2 of Non-POU domain-containing octamer-binding protein OS=Homo sapiens GN=NONO | 1.41 | ± | 0.03 |
| HMGA1 | High mobility group protein HMG-I/HMG-Y OS=Homo sapiens GN=HMGA1 PE=1 SV=3 | 1.37 | ± | 0.04 |
| RPL18A | 60S ribosomal protein L18a OS=Homo sapiens GN=RPL18A PE=1 SV=2 | 1.37 | ± | 0.04 |
| UPF1 | Regulator of nonsense transcripts 1 OS=Homo sapiens GN=UPF1 PE=1 SV=2 | 1.31 | ± | 0.02 |
| RPL26 | 60S ribosomal protein L26 OS=Homo sapiens GN=RPL26 PE=1 SV=1 | 1.29 | ± | 0.01 |
| RPS14 | 40S ribosomal protein S14 OS=Homo sapiens GN=RPS14 PE=1 SV=3 | 1.29 | ± | 0.03 |
| RPL13 | 60S ribosomal protein L13 OS=Homo sapiens GN=RPL13 PE=1 SV=4 | 1.28 | ± | 0.00 |
| RPL3 | 60S ribosomal protein L3 OS=Homo sapiens GN=RPL3 PE=1 SV=2 | 1.25 | ± | 0.01 |
| RRBP1 | Ribosome-binding protein 1 OS=Homo sapiens GN=RRBP1 PE=1 SV=4 | 1.24 | ± | 0.00 |
| CORO1C | Coronin-1C OS=Homo sapiens GN=CORO1C PE=1 SV=1 | 1.24 | ± | 0.01 |
| RPL29 | 60S ribosomal protein L29 OS=Homo sapiens GN=RPL29 PE=1 SV=2 | 1.24 | ± | 0.02 |
| TRIM25 | E3 ubiquitin/ISG15 ligase TRIM25 OS=Homo sapiens GN=TRIM25 PE=1 SV=2 | 1.21 | ± | 0.05 |
| PTMA | Prothymosin alpha OS=Homo sapiens GN=PTMA PE=1 SV=2 | 1.20 | ± | 0.03 |
| RPL6 | 60S ribosomal protein L6 OS=Homo sapiens GN=RPL6 PE=1 SV=3 | 1.19 | ± | 0.04 |
| RPL5 | 60S ribosomal protein L5 OS=Homo sapiens GN=RPL5 PE=1 SV=3 | 1.15 | ± | 0.02 |
| RPL8 | 60S ribosomal protein L8 OS=Homo sapiens GN=RPL8 PE=1 SV=2 | 1.14 | ± | 0.04 |
| RPL7 | 60S ribosomal protein L7 OS=Homo sapiens GN=RPL7 PE=1 SV=1 | 1.14 | ± | 0.03 |
| PABPC1 | Polyadenylate-binding protein 1 OS=Homo sapiens GN=PABPC1 PE=1 SV=2 | 1.12 | ± | 0.03 |
| EIF3A | Eukaryotic translation initiation factor 3 subunit A OS=Homo sapiens GN=EIF3A PE=1 SV=1 | 1.09 | ± | 0.03 |
|  |  |  |  |  |
| Downregulated |  |  |  |  |
| NPC2 | Epididymal secretory protein E1 OS=Homo sapiens GN=NPC2 PE=1 SV=1 | 0.59 | ± | 0.01 |
| CNBP | Cellular nucleic acid-binding protein OS=Homo sapiens GN=CNBP PE=1 SV=1 | 0.65 | ± | 0.02 |
| GSTP1 | Glutathione S-transferase P OS=Homo sapiens GN=GSTP1 PE=1 SV=2 | 0.67 | ± | 0.01 |
| S100A10 | Protein S100-A10 OS=Homo sapiens GN=S100A10 PE=1 SV=2 | 0.71 | ± | 0.02 |
| CLTB | Isoform Non-brain of Clathrin light chain B OS=Homo sapiens GN=CLTB | 0.72 | ± | 0.02 |
| S100A6 | Protein S100-A6 OS=Homo sapiens GN=S100A6 PE=1 SV=1 | 0.72 | ± | 0.02 |
| EEF1D | Isoform 2 of Elongation factor 1-delta OS=Homo sapiens GN=EEF1D | 0.74 | ± | 0.02 |
| PPP2CA | Serine/threonine-protein phosphatase 2A catalytic subunit alpha isoform OS=Homo sapiens GN=PPP2CA PE=1 SV=1 | 0.75 | ± | 0.04 |
| PSMD11 | 26S proteasome non-ATPase regulatory subunit 11 OS=Homo sapiens GN=PSMD11 PE=1 SV=3 | 0.76 | ± | 0.02 |
| MYL6 | Myosin light polypeptide 6 OS=Homo sapiens GN=MYL6 PE=1 SV=2 | 0.76 | ± | 0.01 |
| IPO5 | Importin-5 OS=Homo sapiens GN=IPO5 PE=1 SV=4 | 0.76 | ± | 0.03 |
| TPM4 | Tropomyosin alpha-4 chain OS=Homo sapiens GN=TPM4 PE=1 SV=3 | 0.77 | ± | 0.02 |
| PSMD14 | 26S proteasome non-ATPase regulatory subunit 14 OS=Homo sapiens GN=PSMD14 PE=1 SV=1 | 0.77 | ± | 0.02 |
| CTSD | Cathepsin D OS=Homo sapiens GN=CTSD PE=1 SV=1 | 0.79 | ± | 0.03 |
| UGP2 | UTP--glucose-1-phosphate uridylyltransferase OS=Homo sapiens GN=UGP2 PE=1 SV=5 | 0.80 | ± | 0.02 |
| LGALS7 | Galectin-7 OS=Homo sapiens GN=LGALS7 PE=1 SV=2 | 0.81 | ± | 0.03 |
| CBR1 | Carbonyl reductase [NADPH] 1 OS=Homo sapiens GN=CBR1 PE=1 SV=3 | 0.81 | ± | 0.04 |
| RAD23B | UV excision repair protein RAD23 homolog B OS=Homo sapiens GN=RAD23B PE=1 SV=1 | 0.82 | ± | 0.05 |
| GPI | Isoform 2 of Glucose-6-phosphate isomerase OS=Homo sapiens GN=GPI | 0.85 | ± | 0.03 |
| KRT17 | Keratin, type I cytoskeletal 17 OS=Homo sapiens GN=KRT17 PE=1 SV=2 | 0.85 | ± | 0.02 |
| PLIN3 | Perilipin-3 OS=Homo sapiens GN=PLIN3 PE=1 SV=3 | 0.85 | ± | 0.04 |
| PDIA6 | Isoform 5 of Protein disulfide-isomerase A6 OS=Homo sapiens GN=PDIA6 | 0.86 | ± | 0.05 |
| AARS | Alanine--tRNA ligase, cytoplasmic OS=Homo sapiens GN=AARS PE=1 SV=2 | 0.87 | ± | 0.03 |
| HSPA8 | Heat shock cognate 71 kDa protein OS=Homo sapiens GN=HSPA8 PE=1 SV=1 | 0.88 | ± | 0.02 |
| KRT8 | Keratin, type II cytoskeletal 8 OS=Homo sapiens GN=KRT8 PE=1 SV=7 | 0.88 | ± | 0.05 |
| APEH | Acylamino-acid-releasing enzyme OS=Homo sapiens GN=APEH PE=1 SV=4 | 0.88 | ± | 0.04 |
| SERPINB5 | Serpin B5 OS=Homo sapiens GN=SERPINB5 PE=1 SV=2 | 0.88 | ± | 0.03 |
| KRT19 | Keratin, type I cytoskeletal 19 OS=Homo sapiens GN=KRT19 PE=1 SV=4 | 0.88 | ± | 0.00 |
| P4HB | Protein disulfide-isomerase OS=Homo sapiens GN=P4HB PE=1 SV=3 | 0.88 | ± | 0.03 |
| CDC37 | Hsp90 co-chaperone Cdc37 OS=Homo sapiens GN=CDC37 PE=1 SV=1 | 0.90 | ± | 0.04 |
| HSP90AA1 | Heat shock protein HSP 90-alpha OS=Homo sapiens GN=HSP90AA1 PE=1 SV=5 | 0.90 | ± | 0.03 |
| HSP90B1 | Endoplasmin OS=Homo sapiens GN=HSP90B1 PE=1 SV=1 | 0.91 | ± | 0.02 |
| ACTN4 | Alpha-actinin-4 OS=Homo sapiens GN=ACTN4 PE=1 SV=2 | 0.92 | ± | 0.05 |
| CLTC | Clathrin heavy chain 1 OS=Homo sapiens GN=CLTC PE=1 SV=5 | 0.92 | ± | 0.03 |
| AHNAK | Neuroblast differentiation-associated protein AHNAK OS=Homo sapiens GN=AHNAK PE=1 SV=2 | 0.94 | ± | 0.00 |
| DSP | Desmoplakin OS=Homo sapiens GN=DSP PE=1 SV=3 | 0.95 | ± | 0.04 |
|  |  |  |  |  |
| HPK-(IR)-BS |  |  |  |  |
| Upregulated |  |  |  |  |
| APC | Adenomatous polyposis coli protein OS=Homo sapiens GN=APC PE=1 SV=2 | 2.01 | ± | 0.03 |
| SEC11A | Signal peptidase complex catalytic subunit SEC11A OS=Homo sapiens GN=SEC11A PE=1 SV=1 | 1.66 | ± | 0.04 |
| MTHFD1 | C-1-tetrahydrofolate synthase, cytoplasmic OS=Homo sapiens GN=MTHFD1 PE=1 SV=3 | 1.24 | ± | 0.01 |
|  |  |  |  |  |
| Downregulated |  |  |  |  |
| HACD3 | Very-long-chain (3R)-3-hydroxyacyl-CoA dehydratase 3 OS=Homo sapiens GN=HACD3 PE=1 SV=2 | 0.58 | ± | 0.02 |
| VDAC2 | Voltage-dependent anion-selective channel protein 2 OS=Homo sapiens GN=VDAC2 PE=1 SV=2 | 0.66 | ± | 0.01 |
| HMGB1 | High mobility group protein B1 OS=Homo sapiens GN=HMGB1 PE=1 SV=3 | 0.67 | ± | 0.01 |
| ATP5L | ATP synthase subunit g, mitochondrial OS=Homo sapiens GN=ATP5L PE=1 SV=3 | 0.69 | ± | 0.05 |
| NAP1L1 | Nucleosome assembly protein 1-like 1 OS=Homo sapiens GN=NAP1L1 PE=1 SV=1 | 0.70 | ± | 0.04 |
| DDX5 | Probable ATP-dependent RNA helicase DDX5 OS=Homo sapiens GN=DDX5 PE=1 SV=1 | 0.72 | ± | 0.02 |
| DLD | Dihydrolipoyl dehydrogenase, mitochondrial OS=Homo sapiens GN=DLD PE=1 SV=2 | 0.76 | ± | 0.02 |
| PTGR1 | Prostaglandin reductase 1 OS=Homo sapiens GN=PTGR1 PE=1 SV=2 | 0.78 | ± | 0.05 |
| RTN4 | Isoform 2 of Reticulon-4 OS=Homo sapiens GN=RTN4 | 0.78 | ± | 0.01 |
| LETM1 | LETM1 and EF-hand domain-containing protein 1, mitochondrial OS=Homo sapiens GN=LETM1 PE=1 SV=1 | 0.78 | ± | 0.03 |
| CANX | Isoform 2 of Calnexin OS=Homo sapiens GN=CANX | 0.79 | ± | 0.00 |
| P4HB | Protein disulfide-isomerase OS=Homo sapiens GN=P4HB PE=1 SV=3 | 0.85 | ± | 0.00 |
| CALU | Calumenin OS=Homo sapiens GN=CALU PE=1 SV=2 | 0.87 | ± | 0.02 |
| HSP90B1 | Endoplasmin OS=Homo sapiens GN=HSP90B1 PE=1 SV=1 | 0.88 | ± | 0.03 |
| LDHA | L-lactate dehydrogenase A chain OS=Homo sapiens GN=LDHA PE=1 SV=2 | 0.89 | ± | 0.02 |
| MYH9 | Myosin-9 OS=Homo sapiens GN=MYH9 PE=1 SV=4 | 0.90 | ± | 0.00 |
| PPIB | Peptidyl-prolyl cis-trans isomerase B OS=Homo sapiens GN=PPIB PE=1 SV=2 | 0.90 | ± | 0.03 |
| HSPA5 | 78 kDa glucose-regulated protein OS=Homo sapiens GN=HSPA5 PE=1 SV=2 | 0.91 | ± | 0.00 |
