# Supplementary figures and images for: Low-dose ionizing radiation exposure represses the cell cycle and protein synthesis pathways in in vitro human primary keratinocytes and U937 cell lines
Source: PLoS One. 2018 Jun 18;13(6):e0199117. doi: 10.1371/journal.pone.0199117 (PMC6005503; doi:10.1371/journal.pone.0199117)

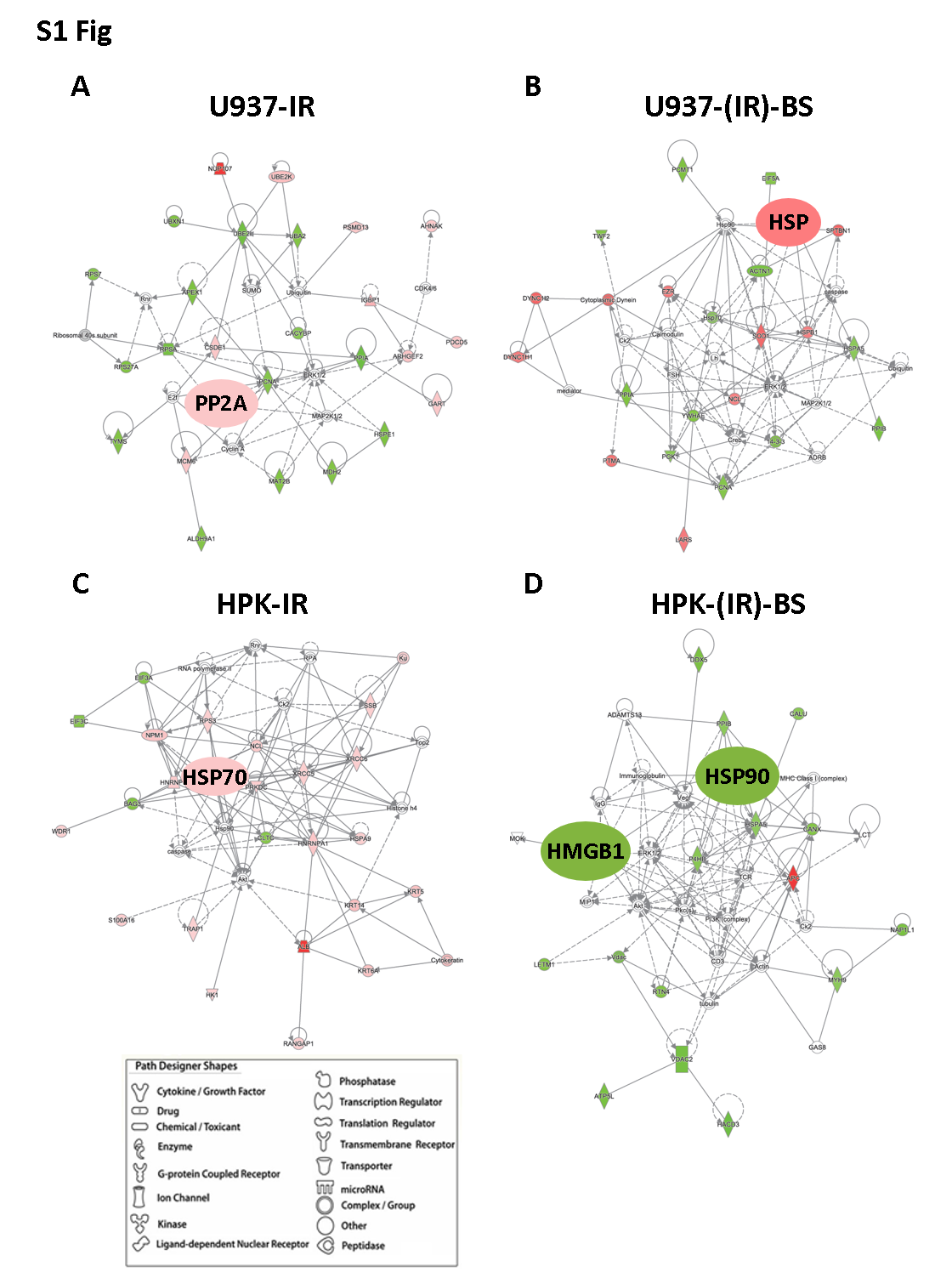

Supplement: S1 Fig — Data were analyzed using IPA (QIAGEN, www.qiagen.com/ingenuity). The IPA network analysis showed direct interactions between differentially expressed molecules in U937 cells and HPKs after the LDIR treatment directly or in the bystander condition. U937-IR cells; (B) U937-(IR)-BS cells; (C) HPK-IR cells; (D) HPK-(IR)-BS cells. Arrows indicate direct interactions between molecules. Lines represent direct (solid lines) and indirect (dashed lines) interactions between molecules. The network with the highest score is shown. Upregulated proteins in the dataset are depicted in pink and downregulated proteins in green. The depth of color indicates the degree of change [72]. (TIF) [file pone.0199117.s003.tif]
